# The widening spectrum of *C9ORF72*-related disease; genotype/phenotype correlations and potential modifiers of clinical phenotype

**DOI:** 10.1007/s00401-014-1251-9

**Published:** 2014-02-04

**Authors:** Johnathan Cooper-Knock, Pamela J. Shaw, Janine Kirby

**Affiliations:** Department of Neuroscience, Sheffield Institute for Translational Neuroscience, University of Sheffield, 385a Glossop Road, Sheffield, S10 2HQ UK

**Keywords:** Amyotrophic lateral sclerosis, Frontotemporal lobar dementia, C9ORF72, G_4_C_2_ expansion, Phenotypic variation, Genetic modifiers

## Abstract

The GGGGCC (G_4_C_2_) repeat expansion in *C9ORF72* is the most common cause of familial amyotrophic lateral sclerosis (ALS), frontotemporal lobar dementia (FTLD) and ALS–FTLD, as well as contributing to sporadic forms of these diseases. Screening of large cohorts of ALS and FTLD cohorts has identified that *C9ORF72*-ALS is represented throughout the clinical spectrum of ALS phenotypes, though in comparison with other genetic subtypes, *C9ORF72* carriers have a higher incidence of bulbar onset disease. In contrast, *C9ORF72*-FTLD is predominantly associated with behavioural variant FTD, which often presents with psychosis, most commonly in the form of hallucinations and delusions. However, *C9ORF72* expansions are not restricted to these clinical phenotypes. There is a higher than expected incidence of parkinsonism in ALS patients with *C9ORF72* expansions, and the G_4_C_2_ repeat has also been reported in other motor phenotypes, such as primary lateral sclerosis, progressive muscular atrophy, corticobasal syndrome and Huntington-like disorders. In addition, the expansion has been identified in non-motor phenotypes including Alzheimer’s disease and Lewy body dementia. It is not currently understood what is the basis of the clinical variation seen with the G_4_C_2_ repeat expansion. One potential explanation is repeat length. Sizing of the expansion by Southern blotting has established that there is somatic heterogeneity, with different expansion lengths in different tissues, even within the brain. To date, no correlation with expansion size and clinical phenotype has been established in ALS, whilst in FTLD only repeat size in the cerebellum was found to correlate with disease duration. Somatic heterogeneity suggests there is a degree of instability within the repeat and evidence of anticipation has been reported with reducing age of onset in subsequent generations. This variability/instability in expansion length, along with its interactions with environmental and genetic modifiers, such as *TMEM106B*, may be the basis of the differing clinical phenotypes arising from the mutation.

## Introduction

**Fig. 1:**
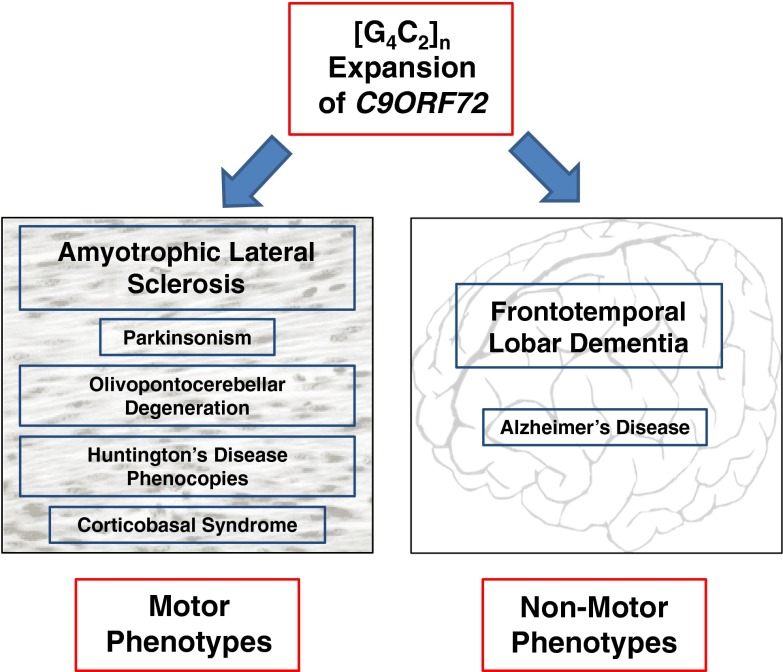
Clinical phenotypes associated with G4C2 repeat expansion of *C9ORF72*: both motor and non-motor phenotypes are associated with *C9ORF72* expansions. The primary phenotype in each case is amyotrophic lateral sclerosis (ALS) and frontotemporal lobar dementia (FTLD). Other phenotypes have been noted in very small numbers of patients: motor phenotypes in this group are parkinsonism, olivopontocerebellar degeneration, corticobasal syndrome and Huntington’s disease phenocopies. The only non-motor phenotype identified in more than a few individuals is Alzheimer’s disease

One of the most interesting features of hexanucleotide repeat expansions of *C9ORF72* is that the associated phenotype is extremely variable and indeed, except in certain pedigrees, the expansion does not appear to be 100 % penetrant [1]. Although it was discovered in patients with amyotrophic lateral sclerosis (ALS) and/or frontotemporal lobar degeneration (FTLD) [2, 3] many of the original studies of cohorts of *C9ORF72*-related patients noted the presence of other clinical phenotypes in probands and family members, at higher frequencies than would be expected by chance [4]. For the purposes of this review, clinical presentations will be divided into motor and non-motor phenotypes within which various recognised symptom complexes will be discussed (Fig. 1). The variability in, and indeed within, clinical presentations of *C9ORF72*-related disease [4] is a source of hope as well as a challenge as it suggests that multiple disease modifiers are at work, each of which is a potential therapeutic target. Understanding what these potential modifiers might be is at an early stage and indeed the number of likely modifiers means that large numbers of cases are going to be needed to illustrate any single effect. The most obvious potential modifier is expansion size, but as yet a clear relationship has not been established in ALS, although there is more indication of a correlation with phenotype in FTLD.

The penetrance of *C9ORF72*-related disease does not appear to differ between ALS and FTLD pedigrees. Identification of repeat expansions in the general population during mutation screening of neurologically normal controls [1, 4] suggests that *C9ORF72* expansions may actually be as common in “controls” as in specific clinical phenotypes, such as corticobasal syndrome (CBS) and sporadic Creutzfeldt–Jakob disease [1, 5]. It has been suggested that these *C9ORF72* “controls” might represent pre-symptomatic disease as estimates suggest that *C9ORF72* expansions are non-penetrant at <35 years, 50 % penetrant by 58 years and fully penetrant at 80 years [5, 6]. Alternatively, the penetrance of the expansion may not reach 100 % even at 80 years, as others have reported *C9ORF72* expansion carriers aged 80 years and over without a clinical phenotype [7]. This implies that the expansions in Alzheimer’s disease cases may be chance occurrences. Based on estimates of prevalence of ALS and FTLD in the UK population being 0.3 and 0.07 %, respectively [8, 9] and the proportion of cases with *C9ORF72* expansions being 10 % for ALS and 13.6 % for FTLD [5], we have calculated the lifetime risk of *C9ORF72*-related disease to be approximately 0.04 %. There is, therefore, a 15-fold difference between the estimated frequency of *C9ORF72* repeat expansions in controls of up to 0.6 % [4], and the lifetime risk of *C9ORF72*-related disease. This difference is perhaps too large to be explained by premature death of expansion carriers from other causes, although this remains to be verified. The variable penetrance also raises the possibility that *C9ORF72* expansions may be a risk factor for disease and may not be capable of producing disease in isolation. There is a higher than expected coincidence of repeat expansions found in individuals carrying other genetic variants of ALS, so-called oligogenic inheritance [10], suggesting that another ‘hit’ may be necessary for clinical disease. Oligogenic inheritance has also been reported in FTLD, with the *C9ORF72* expansion being identified in patients carrying *GRN* mutations [11, 12].

## *C9ORF72* in ALS and other motor phenotypes

### Epidemiology

Whilst the majority of cases of ALS are sporadic, data show that 5–10 % of ALS cases show clear autosomal dominant inheritance [13], whilst twin studies estimate that the genetic heritability of disease is between 0.38 and 0.78 [14]. Over 20 loci have been identified, with the genes identified at 17, the most common causes being mutations of *C9ORF72*, *SOD1*, *TARDBP* and *FUS* [15].

Screening for the *C9ORF72* expansion in ALS cohorts worldwide has identified this mutation as the most common genetic cause of ALS in Caucasians, and the proportion of patients affected is significant in this complex neurodegenerative disease. In the Northern England population, *C9ORF72* expansions are present in 43 % of ALS cases with an identifiable family history and in 7 % of apparently sporadic cases [4]. However, this frequency varies globally: *C9ORF72* expansions were found in 61 % of familial ALS and 19 % of sporadic ALS patients in Finland [5], 22 % of familial ALS cases in Germany [5] and only 3.4 % in Japan [16]. The apparent correlation of expansion frequency with distance from Scandinavia is consistent with a suggested common founder rather than repeated *de novo* occurrence. Indeed the *C9ORF72* expansion was identified through study of a risk haplotype at the 9p21 locus [17]. Following the screening of numerous populations for the *C9ORF72* expansion, it has been shown to occur in the presence of the same haplotype in all populations considered, or with the A-allele of rs389942, which is associated with the haplotype [18], including populations which might be considered relatively genetically different from Scandinavia such as Japan [16]. Data analysis of a 42-SNP haplotype from carriers of the expansion in Europe, USA and Australia led to the hypothesis that there was a common founder carrying the pathogenic mutation approximately 100 generations ago in Northern Europe and this expansion then spread across the world [5]. A European study using an 82-SNP haplotype suggests the expansion is much older, with the common founder arose over 251 generations ago [19]. As the disease has a late age of onset, this means it is not subject to reproductive pressure. However, other studies have contradicted the founder effect, showing no evidence of recent shared ancestry in cases identified within the South-east region of the UK and the presence of the expansion in a patient homozygous for the non-risk G-allele [1, 20].

The origin of the expansion becomes even more intriguing if it is considered whether it is the expansion itself or a propensity for the region to expand that is inherited. One of the initial studies of *C9ORF72* noted that the risk haplotype was associated with an increased number of repeats even in controls [3]. It was determined, in a group of controls, that longer repeat lengths were associated with the rs389942 A-allele and that over 10 repeats was associated with inter-generational changes in repeat length [1].

### *C9ORF72*-related ALS phenotypes

ALS is a neurodegenerative disease defined clinically as the loss of upper and/or lower motor neurons. It is a disease of ageing: peak age of onset is between 50 and 70 years [21]. Progression is relentless and death usually results from respiratory failure, between 3 and 5 years after onset of symptoms [22]. However, the ALS phenotype is notably variable; approximately 20 % of patients survive longer than 5 years [23] and cases have been reported in teenagers and the very elderly. The disease usually starts in one area and spreads in an anatomically contiguous fashion throughout the motor system [24]. Typically this involves insidious progression of weakness from one limb or the bulbar muscles; rarely does the disease simultaneously start in multiple areas or in the respiratory muscles.

Broadly, the ALS phenotype associated with G_4_C_2_ repeat expansion of *C9ORF72* is representative of the whole clinical spectrum of ALS [4, 25, 26]. However, within the broad range of phenotypes, bulbar onset has been found to be more frequently associated with *C9ORF72*-related ALS, than with non-*C9ORF72* ALS [4, 27–29]; and as would be expected from the association of *C9ORF72* with FTLD, there is a greater incidence of dementia or a family history of dementia in *C9ORF72*-related ALS, than in non-*C9ORF72* ALS [4, 25, 29–32]. In addition, there is evidence of an earlier age of onset in *C9ORF72*-related ALS compared to non-*C9ORF72* ALS cases [30, 31] though not all cohorts reached significance [4, 31]. However, in a Belgian cohort, where specifically familial cases with *C9ORF72*-related ALS were compared to non-*C9ORF72* familial ALS cases, the age of onset was later in *C9ORF72*-related familial ALS [27]. In contrast, there were no differences between *C9ORF72*-related and non-*C9ORF72* sporadic ALS cases. Finally, evidence suggests that *C9ORF72*-related ALS has a shorter survival than non-*C9ORF72* ALS [4, 27–32]. Some of the discrepancies between these reports may be explained by different cohorts being used in the comparisons (e.g. all *C9ORF72*-related ALS v all non-*C9ORF72* ALS as opposed to familial *C9ORF72*-related ALS v familial non-*C9ORF72* ALS) and due to the extensive variability in the non-*C9ORF72* ALS phenotype. However, gender may also play a role as male *C9ORF72*-related ALS cases have been reported to have a younger age of onset than non-*C9ORF72* ALS cases [33].

To address the variability of non-*C9ORF72* ALS, one study compared a large cohort of *C9ORF72*-related ALS cases directly with patients carrying other genetic variants [34]. This showed that *C9ORF72*-related cases had a significantly higher incidence of bulbar onset compared to ALS cases with mutations in *SOD1*, *TARDBP*, *FUS* and other familial ALS cases, as well as a greater association with FTLD. Disease duration of *C9ORF72*-related ALS was significantly shorter than in patients with mutations in *SOD1*, *TARDBP* or other familial ALS cases, but did not differ from *FUS* cases, whilst there was an older age of onset in *C9ORF72*-related ALS compared to *SOD1* and *FUS*-related ALS, but not when compared with *TARDBP* and other familial ALS cases.

### Other motor phenotypes

#### Parkinsonism

What is most clear about the *C9ORF72*-related ALS phenotype compared to other ALS cases, whether familial or sporadic, is an association with other neurological phenotypes both motor and extramotor [4, 34]. Most prominently this includes FTLD and a number of other non-motor phenotypes (which will be discussed further in the section on “FTLD and other dementia and psychosis-related phenotypes”). In addition to FTLD, we and others also noted an association with other motor phenotypes including parkinsonism; by which we refer to the clinical presentation rather than confirmed Parkinson’s disease (PD) with α-synucleinopathy. It is clear that parkinsonism is over-represented compared to chance in *C9ORF72*-related ALS cases and their families [3, 4, 35, 36]. Despite this, a number of studies have failed to find *C9ORF72* expansions in patients with a clinical diagnosis of pure PD [37–39] or only very rarely and often in patients with a family history of ALS/FTLD [40, 41] perhaps suggesting a pathogenesis distinct from classical idiopathic PD. Focusing on the neuropathology of these cases, added some clarity to this point. Whilst there was no evidence for over-representation of *C9ORF72* expansions in a cohort of PD patients with confirmed α-synucleinopathy, neurodegeneration was shown in the substantia nigra of *C9ORF72*-related ALS cases [40]. Therefore, it is suggested that whilst *C9ORF72* expansions are not a cause of classical PD, the neuropathology associated with *C9ORF72*-related ALS can affect the substantia nigra and cause parkinsonism. Thus, *C9ORF72* expansions can affect multiple brain areas which lead to different clinical consequences.

#### Multiple sclerosis

Another phenotype associated with *C9ORF72*-related ALS was demyelination [4]. To further investigate this, a cohort of multiple sclerosis (MS) cases was screened but no *C9ORF72* expansions were identified. However, in a small number of prospectively identified cases with MS who subsequently developed ALS, there was a significantly higher than expected number of *C9ORF72* expansions [42]. Rather than *C9ORF72* expansions causing MS it is suggested MS increased the penetrance of the *C9ORF72* expansion, and this was supported by the fact that *C9ORF72*-related ALS was more rapidly progressive in the patients with a previous history of MS.

#### Other motor phenotypes screened or associated with for C9ORF72 expansions

With the widening clinical phenotypes associated with ALS, several other neurodegenerative disease cohorts have been screened to determine if they are associated with the *C9ORF72* repeat expansion. Whilst ALS is defined as the loss of both upper and lower motor neurons, primary lateral sclerosis (PLS) is characterised by the degeneration of only upper motor neurons whilst the loss of only lower motor neurons is defined as progressive muscular atrophy (PMA). These three disorders are considered to form part of a spectrum of disease. However, screening for the *C9ORF72* expansion found the expanded repeats at relatively low frequencies in the PMA and PLS cohorts, at 1.6 and 0.9 %, respectively [43], and in a smaller cohort of PMA, PLS and progressive bulbar palsy patients, no expansions were found [44]. Thus, *C9ORF72* expansions appear to be more commonly associated with an ALS phenotype within this spectrum of disease.

Hereditary spastic paraplegia (HSP) describes a group of over 50 genetically heterogeneous diseases which are characterised by a progressive dying back of the distal axons of upper motor neurons, leading to lower limb spasticity, pyramidal distribution weakness in the lower limbs and brisk reflexes. To establish if *C9ORF72* might be a modifier of the HSP phenotype, a Danish cohort of 182 HSP cases was screened for the repeat expansion [45]. However, no mutations were identified. The mutation was not associated with any cases of corticobasal syndrome or progressive supranuclear palsy (PSP) in Italy or the United States [46, 47] but was identified in a Danish patient with CBS [48]. In addition, where ALS + CBS and ALS + PSP were the clinical diagnosis, *C9ORF72* expansions were identified [49]. Finally, a pathological characterisation of *C9ORF72*-FTLD cases identified an individual with FTLD-tau and corticobasal degeneration [50].

In the Danish cohort of dementia cases, which included the *C9ORF72*-related CBS case, *C9ORF72* expansions were also found in one patient clinically diagnosed with olivopontocerebellar degeneration (OPCD) (where his father had ALS) and one patient with atypical Parkinsonian syndrome (APS) [48]. The range of motor phenotypes associated with *C9ORF72* expansions was widened further with expansions found in multiple cases of a cohort of Huntington disease-like syndromes and “other neurodegenerative diseases” (excluding ALS and FTLD) [1]. The association of *C9ORF72* with HD-like symptoms was supported by 1.95 % cases within a cohort of HD phenocopies being positive for the expansion [51]. Thus, the *C9ORF72* expansion is associated with additional motor phenotypes, outside of the ALS spectrum, albeit at low frequencies.

## *C9ORF72* in FTLD and other dementia and psychosis-related phenotypes

### Epidemiology

Whilst the majority of cases of FTLD are sporadic, a body of evidence shows that approximately 13 % of FTLD cases show clear autosomal dominant inheritance. However, up to 40 % of cases have an additional member of the family with the disease, though whether this is a contribution of genes or environment or both remains to be established [52].

Mutations in *MAPT*, *CHMP2B*, *VCP*, *GRN*, *TARDBP* and *FUS* have been previously published as genetic causes of autosomal dominant FTLD. However, with the identification of the *C9ORF72* repeat expansion as a cause of FTLD and ALS–FTLD [2, 3], many cohorts of FTLD cases have been screened to establish the frequency of this gene in FTLD. A consistent finding is that the *C9ORF72* expansion is the most frequent mutation associated with familial cases, accounting for 25.1 % of familial FTLD [5]. In addition, the *C9ORF72* mutation is also found in 6 % of sporadic FTLD cases, though there is a wide variation in frequency across different populations, with the highest frequencies in geographically isolated populations such as Finland (21 %) [53] and the lowest frequency found in Holland (2.2 %) [5]. The mutation is most frequently found in Caucasian populations, although the expansion has recently been shown in two Chinese cases, one with sporadic FTLD and another with familial ALS–FTLD [54]. This is in contrast to ALS, where *C9ORF72* carriers are seen in Native American, Hispanic, Middle Eastern and Asian populations [5].

As discussed earlier, the expansion is suggested to have arisen over 100 generations ago in Northern Europe, based upon analysis that suggested expansion carriers all carried the same risk haplotype [5]. However, as with ALS, this is not always the case [11]. Ferrari and colleagues report four cases which carried the surrogate risk haplotype marker rs3849942 A-allele, but did not carry the complete 42 SNP risk haplotype. This finding is most likely to be due to the ethnically diverse background of these patients from the USA and the effect of recombination events throughout the preceding generations.

In addition, what is particularly interesting about the chromosome 9p21 region is that if expansion carriers are excluded, the locus still shows significant linkage to the ALS phenotype, suggesting a further genetic risk factor may be associated with the same region. This may perhaps point to the presence of another expansion [55]. Further sequencing and analysis of the region will be required to elucidate the basis of this linkage finding.

### *C9ORF72*-related FTLD phenotypes

FTLD is the second most common form of degenerative dementia after Alzheimer’s disease to occur in individuals younger than 65 years old, and accounts for 5–15 % of all dementia cases. Survival is on average 7 years from onset. It can be subdivided according to the presenting features into behavioural variant FTLD (bvFTLD), where there is a change in the patients behaviour associated with a progressive deterioration of personality, usually beginning with hallucinations, delusions, disordered thinking or paranoia, or primary progressive aphasia (PPA). PPA can be further subdivided into progressive non-fluent aphasia (PNFA) where patients have difficulties with word retrieval, non-fluent speech patterns and a progressive loss of speech, and semantic dementia (SD), where there is a loss of memory regarding the understanding of words and objects. In addition, FTLD can be associated with extrapyramidal movement disorders, such as parkinsonism or corticobasal syndrome as well as amyotrophic lateral sclerosis/motor neurone disease (FTLD–ALS/FTLD–MND), which will be discussed later.

The predominant FTLD phenotype associated with *C9ORF72* mutations is that of bvFTLD. The high incidence of this variant in the *C9ORF72* expansion carriers was reported in the initial publications [2, 3]. Subsequent screening of FTLD cohorts across the UK, Europe, America and Australia identified bvFTLD as consistently more prevalent in *C9ORF72*-related FTLD than non-*C9ORF72* FTLD cases [6, 11, 20, 25, 26, 35, 46, 47, 53, 56–60]. The percentage of bvFTLD within *C9ORF72*-related FTLD varied within the cohorts, but it was always higher by 12–19 % [6, 53, 59]. Within the *C9ORF72* bvFTLD cases there were a significant number who presented with psychosis, most commonly hallucinations and delusions [47, 59]. This is exemplified in a UK study where a third of the *C9ORF72*-related FTLD cases presented with psychosis, compared to only 4 % in the non-*C9ORF72* cases [59]. Whilst bvFTLD was the most common FTLD variant associated with *C9ORF72* expansion, PNFA was the second most common FTLD variant associated with *C9ORF72* expansions, although the frequency of PNFA did not differ between carriers and non-carriers of the expansion. SD has been seen more rarely associated with *C9ORF72* expansions [58, 59, 61].

The clinical phenotype of *C9ORF72*-related FTLD has also been compared to FTLD cases carrying mutations in *GRN* and *MAPT*. In an early study, no clinical differences were seen between *C9ORF72*, *GRN* and *MAPT* cases, although neuroimaging showed predominant frontal atrophy was more common in the *C9ORF72*-related cases. A subsequent study showed that the *C9ORF72*-related FTLD cases showed an earlier age of onset than *GRN* mutation carriers, whilst survival was similar, and whereas bvFTLD was most commonly associated with *C9ORF72* expansions, PNFA was more commonly associated with *GRN* mutations [60]. Within a cohort of bvFTLD the relative frequencies of these three genetic variants showed that in familial cases, the frequency of *C9ORF72* was similar to *MAPT* mutations (14.7 and 12.7 %, respectively) but *GRN* mutations were of lower frequency (6.8 %) [35]. In sporadic bvFTLD cases, however, the frequency of these three mutations in the cohort was similar, at 3–4 %.

### Other dementia and psychosis-related phenotypes

#### Alzheimer’s disease

Initial screening of Alzheimer’s disease (AD) patients either failed to find any *C9ORF72* expansions or suggested that the cases were misdiagnosed FTLD cases [5, 47, 62]. Indeed, pathological studies of these initial Alzheimer’s disease cases identified none of the p62-positive inclusions in hippocampus and cerebellum which are considered pathognomonic for *C9ORF72* disease [41, 63]. However, subsequent screening of AD cohorts has identified several cases with *C9ORF72* expansions, including individuals with autopsy confirmed AD [64–66]. The cases were also shown to carry the risk A-allele at rs389942. In addition, it was found that these *C9ORF72*-related AD cases have a later age of onset, at 77.8 years, compared to the rest of the cohort of over 1,000 definite or probable AD cases [66, 67]. However, no association of *C9ORF72* with the APOE ε4 status was identified [65, 66]. Therefore, the *C9ORF72* expansion is associated with AD at low frequencies (<1 %), but notably more often than in healthy controls [64–66].

#### Other dementia-related disorders screened or associated with for C9ORF72 expansions

Expansions of *C9ORF72* have also been identified in single cases of dementia with Lewy bodies [68] and sporadic Creutzfeldt–Jakob disease [1], although the former patient is proposed to suffer from a FTLD TDP-proteinopathy that mimics Lewy body dementia. Interestingly, whilst psychosis is highly associated with *C9ORF72*-related bvFTLD, no *C9ORF72* expansions were seen in a cohort of 192 schizophrenia cases [69].

## ALS–FTLD

ALS and FTLD have been linked both through families presenting with ALS, FTLD or both diseases. It was through studying these families that the Chr9p21 locus and *C9ORF72* as a genetic cause of ALS and FTLD was identified [2, 3]. Several other genes associated with ALS have subsequently been found, albeit less commonly, in FTLD (*TARDBP*, *FUS*) and vice versa (*CHMP2B*). However, *C9ORF72* provides the strongest link between the two disorders. Whilst previously familial ALS or familial FTLD was classified if there was a close family member with the disease, the incidence of familial disease increases if both ALS and dementia are included [4]. Screening of ALS–FTLD cases has shown that *C9ORF72* expansion is the most common cause of familial ALS–FTLD, accounting for the majority (sometimes all) of the ALS–FTLD cases screened [25, 26, 56]. Individuals can present with ALS or FTLD first and then go on to develop the other disease manifestation later. Clinically and pathologically patients possess both classical ALS and FTLD symptoms and neuropathology. The molecular basis for this disease specification is currently unknown, although work is beginning to elucidate the effect of the expansion length and other genetic and environmental modifiers on *C9ORF72* disease.

## Clinical phenotype specification: role of expansion length

In a repeat expansion-related disorder the most obvious candidate for a genetic modifier is repeat number. Measurement of the G_4_C_2_ repeat length initially proved technically challenging as it is not amenable to PCR-based sequencing. However, a number of groups have now optimised Southern hybridisation-based techniques [1, 70, 71] which allow sizing of the expansion. So far, in a pure ALS group, no aspect of phenotype has been shown to significantly correlate with the length of the expansion regardless of the tissue tested [71, 72]. However, in FTLD, van Blitterswijk et al. [71] revealed a direct correlation between repeat size in the frontal cortex and age of onset, and a threshold repeat size in the cerebellum associated with reduced survival. The work also established that the repeat length in the cerebellum was shorter than in other CNS areas (mean of approximately 1,667 repeat units compared to approximately 5,250 repeat units in the frontal cortex). It is hypothesised that repeat expansions can increase in size through a human lifetime resulting in significant somatic heterogeneity [73] and therefore, perhaps the minimum repeat length in the CNS is more reflective of the germline repeat number. If so, the expansion length in the cerebellum may best represent the repeat length which initiated the disease pathogenesis; and the correlation with age of onset in frontal cortex may simply reflect the patient’s age. In this context it is interesting, but not conclusive, to note that the same study found smaller repeat lengths in blood from two out of three unaffected expansion carriers compared to their affected relatives. A smaller study identified a positive correlation between repeat length and age of onset in *C9ORF72*-related patients with a variety of neurodegenerative phenotypes including FTLD, ALS and Alzheimer’s disease [1] but as described above, this may simply reflect the individual’s age at the time of sampling. It has also been shown that the length of the non-expanded allele is not a disease modifier in *C9ORF72*-related or non-*C9ORF72* ALS or FTLD [71, 74, 75].

One of the significant questions is whether there is evidence of anticipation in *C9ORF72*-related disease. Benussi and colleagues [6] reported on *C9ORF72*-related FTLD families which showed evidence of anticipation with a mean difference in age of onset between the parent and offspring of 9.8 years; This was also seen by Chio et al. [76], with age of onset 7 years earlier in the subsequent generation in Italian ALS cases. However, whether this related to expansion size is still to be determined, perhaps through Southern blotting analysis of parents and offspring DNA.

**Fig. 2:**
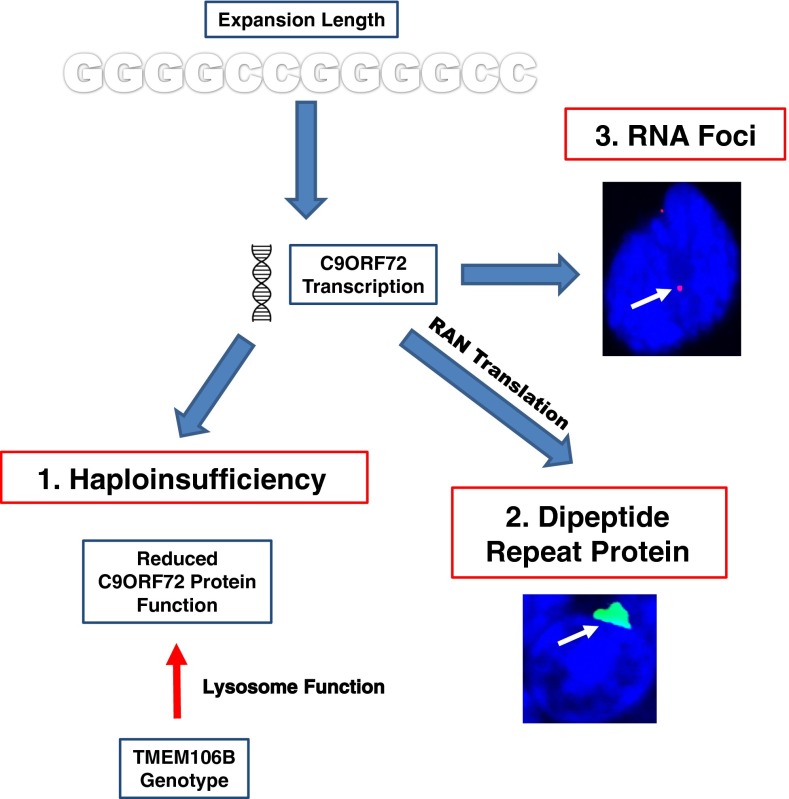
Proposed pathogenic mechanisms and modifiers in *C9ORF72* disease. Three prominent mechanisms of pathogenesis have been proposed in *C9ORF72* disease: (*1*) Haploinsufficiency, (*2*) RAN translation of the expansion to form dipeptide repeats and (*3*) the formation of toxic RNA foci. Poly-(Gly-Ala)- dipeptide repeat protein is shown (*stained in green*, *arrowed*) aggregated in the cytoplasm of a cerebellar granule cell from a *C9ORF72*-ALS patient. RNA foci containing the G4C2 repeat sequence are shown (*stained red*, *arrowed*) within a fibroblast obtained from a *C9ORF72*-ALS patient. There is some evidence that the repeat length is a modifier of the disease phenotype which would be consistent with mechanisms (*2*) and (*3*). However, case reports suggest that disease phenotype is not directly proportional to the number of affected alleles which goes against mechanism (*1*). The function of the C9ORF72 protein is unknown, but a role in membrane trafficking has been proposed which is consistent with the modifier effect of the TMEM106B genotype: both C9ORF72 and TMEM106B are postulated to be involved in lysosome function

In summary, a clear relationship between *C9ORF72* expansion size and disease severity has not yet been identified, particularly in ALS. Comparison with the literature on myotonic dystrophy type I (DM1) is informative on this point. Similar to *C9ORF72*-related disease, DM1 is caused by a repeat expansion in a non-coding region of DMPK. In DM1, correlation between repeat size and phenotype is apparent, but only when a large number of cases (>100) are considered [77]. The reason for this is thought to be the presence of other disease modifiers, the effect of which must be averaged out before a genotype–phenotype correlation is identifiable. Clinically this is relevant because it means that measurement of repeat length could never serve as a prognostic biomarker. As yet the studies of expansion length in *C9ORF72*-ALS have only included relatively small numbers of patients and so there is still a case to answer. Work on the pathogenic mechanism in *C9ORF72* disease appears to be settling on a gain-of-function toxicity mediated either by RNA foci formed from the repeat sequence [2] or dipeptide repeat protein formed by repeat associated non-ATG (RAN) translation [78, 79]. Numbers of RNA foci have been correlated with pathogenic severity in cell models [80, 81], and in tissue from FTLD cases [82]. RAN-translated dipeptide repeat protein appears to be toxic in a cell model [83], but levels of the aberrantly translated protein observed do not correlate with neurodegeneration in autopsy material [34]. If, as postulated, pathogenesis is mediated in a gain-of-function manner then expansion size would be expected to modify the disease phenotype (Fig. 2).

Other than disease severity it is also interesting to ask whether expansion size has an effect on perhaps the most noticeable source of variability in the *C9ORF72* disease phenotypes: the primary group of neurons affected. Currently, work has been limited to comparing *C9ORF72*-ALS and *C9ORF72*-FTLD cases. One study suggested that longer repeat sizes in blood are associated with ALS as opposed to FTLD [72], but others suggested that there is no difference between the two subtypes [1, 71]. However, the largest group size with a pure phenotype in either of these studies was 38 patients; larger numbers will be required to verify either conclusion.

Finally, the G_4_C_2_ expansions have been described as pathogenic if they are over 30 repeats. However, relatively small repeat sizes are reportedly pathogenic in FTLD [84] and the clinical phenotype of ALS cases with 20–30 repeats are similar to those with over 30 repeats [85]. Therefore, the minimum number of repeats required for pathogenicity may be smaller than the currently adopted cut-off size of 30 repeats. If true, this has an impact on another hypothesised pathogenic mechanism in *C9ORF72* disease: haploinsufficiency. Reduced expression of *C9ORF72* mRNA is seen in the presence of the expansion [2]. However, it has been demonstrated that small expansions of approximately 50 repeats do not reduce transcription [86] possibly because smaller expansions do not lead to hypermethylation of a CpG island 5′ to the repeat sequence [87]. If smaller repeat lengths are pathogenic, then haploinsufficiency is not the responsible mechanism. Another observation not consistent with haploinsufficiency comes from two patients with expansions of both *C9ORF72* loci; one a homozygote [16] and the other a compound heterozygote [86]. Both cases suffered FTLD but neither phenotype was outside the usual phenotypic spectrum. This is not consistent with a pure haploinsufficiency model which would predict a disease severity in proportion to the number of involved alleles. It remains to be seen whether haploinsufficiency is a disease modifier, in particular we await the arrival of a specific antibody to accurately detect protein levels of C9ORF72, but evidence is growing that it cannot be the sole or even the primary mechanism in *C9ORF72* disease (Fig. 2).

## Clinical phenotype specification: other potential disease modifiers

The striking variability in the phenotype associated with the *C9ORF72* expansion suggests that multiple modifiers may exist, which may be genetic or environmental. Each identified modifier is a potential therapeutic target and, therefore, understanding factors influencing the phenotype of *C9ORF72* disease is likely to be an important avenue of research in the near future. In addition to the expansion length (described above), a number of other modifiers have been investigated, including TMEM106B genotype.

### TMEM106B genotype

A genome-wide association study initially identified single nucleotide polymorphisms (SNPs) in TMEM106B as risk factors for FTLD with TDP-43 positive pathology (which is found in *C9ORF72*-related FTLD cases amongst other subtypes) [88]. The protein product of TMEM106B is localised to the lysosome. Its function is as yet only beginning to be understood, but it has been suggested that the protective isoform is expressed at a lower level because of increased protein degradation mediated via altered glycosylation [89].

The haplotype associated with higher risk of FTLD-TDP, more particularly the major, or T, allele of rs1990622, has recently been investigated in the context of *C9ORF72*-related disease [90]. The major allele is significantly associated with FTLD presentations of *C9ORF72*-related disease and is associated with an earlier age of onset in *GRN*-related FTLD. However, in *C9ORF72*-FTLD, the major allele is associated with a later age of onset and death. There is no effect on the phenotype in *GRN*-negative non-*C9ORF72* FTLD-TDP patients. This fascinating complexity suggests an interaction between the *C9ORF72* expansion and TMEM106B genotype and suggests both proteins may have similar function. Interestingly, it has been suggested that the *C9ORF72* protein is structurally associated with the DENN family of proteins [91] which are implicated in the regulation of membrane trafficking required for lysosome function (Fig. 2).

Interestingly, in contrast to the C9ORF72-FTLD findings; it has been shown that neither TMEM106B allele is significantly associated with a C9ORF72-related ALS presentation [92]. Why the TMEM106B genotype modifies the risk of one phenotype and not the other is unknown, but this suggests that the mechanism of neurotoxicity may be different in each case.

### Other modifiers

As mentioned above, we have suggested that multiple sclerosis influences the penetrance and phenotype of *C9ORF72*-related ALS patients. Moreover, we showed that, in contrast to non-*C9ORF72* ALS cases, *C9ORF72*-related ALS cases showed a reduction in CSF levels of the cytokine CXCL10 [42] which is reportedly neuroprotective in ALS [93]. Neuroinflammation and activation of microglia have been detected pathologically [94] and in imaging studies [95] of ALS patients. It is thought that the activity of non-neuronal cells may be key determinants of disease propagation through the CNS [96], if not in disease initiation directly.

## Conclusion

Identification of the *C9ORF72* repeat expansion has established the most common cause of ALS, FTLD and ALS–FTLD. Recent screening of related clinical phenotypes has extended the disease spectrum in which the *C9ORF72* expansion is found, including Alzheimer’s disease and parkinsonism. As Southern blotting is used to more accurately size the repeat in larger cohorts of cases, and in multiple tissues, correlations between the expansion length and clinical characteristics may be revealed. Finally, given the frequency of the expansion in both ALS and FTLD, this population will provide an ideal group to elucidate the role of genetic and environmental modifiers contributing to the heterogeneity of disease.
